# A 3-Year Single Center Experience With Left Atrial Pressure Remote Monitoring: The Long and Winding Road

**DOI:** 10.3389/fcvm.2022.899656

**Published:** 2022-06-13

**Authors:** Attilio Restivo, Domenico D'Amario, Donato Antonio Paglianiti, Renzo Laborante, Giuseppe Princi, Luigi Cappannoli, Antonio Iaconelli, Mattia Galli, Nadia Aspromonte, Gabriella Locorotondo, Francesco Burzotta, Carlo Trani, Filippo Crea

**Affiliations:** ^1^Department of Cardiovascular and Pulmonary Sciences, Catholic University of the Sacred Heart, Rome, Italy; ^2^Department of Cardiovascular Medicine, Fondazione Policlinico Universitario A. Gemelli IRCCS, Rome, Italy; ^3^Department of Cardiology, Maria Cecilia Hospital, GVM Care and Research, Cotignola, Italy

**Keywords:** heart failure, hemodynamic remote monitoring, device in heart failure, digital health, telemonitoring, remote care technologies

## Abstract

**Background:**

Despite continuous advancement in the field, heart failure (HF) remains the leading cause of hospitalization among the elderly and the overall first cause of hospital readmission in developed countries. Implantable hemodynamic monitoring is being tested to anticipate the clinical exacerbation onset, potentially preventing an emergent acute decompensation. To date, only pulmonary artery pressure (PAP) sensor received the approval to be implanted in symptomatic heart failure patients with reduced ejection fraction. However, PAP's indirect estimation of left ventricular filling pressure can be inaccurate in some contexts.

**Methods:**

The VECTOR-HF study (NCT03775161) is examining the safety, usability and performance of the V-LAP system, a latest-generation device capable of continuously monitoring left atrial pressure (LAP). In our center, five advanced HF patients have been enrolled. After confirmation of the transmitted data reliability, LAP trends and waveforms have guided therapy optimization. The aim of this work is to share clinical insights from our center preliminary experience with V-LAP application.

**Results:**

Over a median follow-up time of 18 months, LAP–based therapy optimization managed to reduce intracardiac pressure over time and no hospital readmission occurred. This result was paralleled by an improvement in both functional capacity (6MWT distance 352.5 ± 86.2 meters at baseline to 441.2 ± 125.2 meters at last follow-up) and quality of life indicators (KCCQ overall score 63.82 ± 16.36 vs. 81.92 ± 9.63; clinical score 68.47 ± 19.48 vs. 83.70 ± 15.58).

**Conclusion:**

Preliminary evidence from V-LAP application at our institution support a promising efficacy. However, further study is needed to confirm the technical reliability of the device and to exploit the clinical benefit of left-sided hemodynamic remote monitoring.

## Introduction

Heart failure (HF) is a complex clinical syndrome, affecting millions of patients worldwide (1). Despite recent advancements in treatment options, the HF-related hospitalization rate remains unacceptably high and is associated with a worse prognosis (2).

In this scenario, facilitating the journey of a patient through remote monitoring could be intended as a significant step forward. Whether non-invasive monitoring is still lacking conclusive evidence of benefit, the efficacy of implantable hemodynamic monitoring is currently being tested.

Fluid overload represents a key driver for worsening HF and hospitalizations. Its detection and assessment at an early stage is the main task for good management of HF, but it is currently done sub-optimally. The rationale beneath the application of wireless hemodynamic monitoring lies in the pathophysiological demonstration that clinical manifestations of congestive HF appear late in the progression to acute decompensation, whereas intracardiac pressures rise gradually and can anticipate, even by weeks, the onset of the symptoms, offering the opportunity to provide an accurate assessment of incipient pulmonary congestion (3–5). The transition from reactive management, in response to late signs and symptoms occurrence, to a proactive attitude, empowered to timely optimize and anticipate the treatment, thus maintaining normal filling pressures, could prevent acute decompensation.

The recent availability of miniaturized implantable sensors, able to provide a real-time measurement of the intracardiac pressures, may allow treatments to be early enhanced and hemodynamic-tailored to each patient's need. Several implantable monitoring devices have been developed in the past, but the CardioMEMS^TM^ System (Abbott, Sylmar, California) is the only one approved by the regulatory agencies (6). It is a miniaturized sensor implanted in a branch of the pulmonary circulation after a right heart catheterization (RHC) procedure, able to detect pulmonary artery pressures (PAP) and to transmit these data remotely to clinicians for review. The CHAMPION (6) and GUIDE-HF (7) trials tested the efficacy of this approach, but they obtained uncertain findings, resulting in the current IIb (level B) class of recommendation for PAP monitoring by the European Society of Cardiology (ESC) and American Heart Association (AHA)/American College of Cardiology (ACC)/Heart Failure Society of America (AHA/ACC/HFSA) guidelines (8, 9). Several reasons could explain the disappointing results of the PAP monitoring approach. A possible explanation may be the mismatch between right and left intracardiac pressures, affecting the reliability of PAP to estimate the left ventricular filling pressure (LVEDP) (10).

The introduction of a novel class of implantable sensors, able to measure left atrial pressure (LAP), may overcome these limitations (11). In our center, 5 patients were implanted with this sensor as a part of a multicenter trial (12).

## Method

The V-LAP™ system (Vectorious Medical Technologies, Ltd, Tel-Aviv, Israel) is the latest-generation device, capable of monitoring the LAP directly with an intracardiac leadless sensor and transmitting data (values and waveforms) wirelessly to an external reader (11). Once it is percutaneously implanted through the interatrial septum, patients are required to collect daily measurements with an external unit; data are then transferred to the clinicians *via* a secured cloud-based system. The safety, usability, and performance of this new technology are currently being tested in the V-LAP™ Left Atrium Monitoring system for Patients with Chronic systolic and Diastolic Congestive Heart Failure (VECTOR-HF) trial (ClinicalTrials.gov Identifier: NCT03775161). After 3 months of the implantation, the pulmonary capillary wedge pressure (PCWP), measured during an RHC procedure, was compared with the LAP, and assessed by V-LAP, according to the study design.

**Figure 1:**
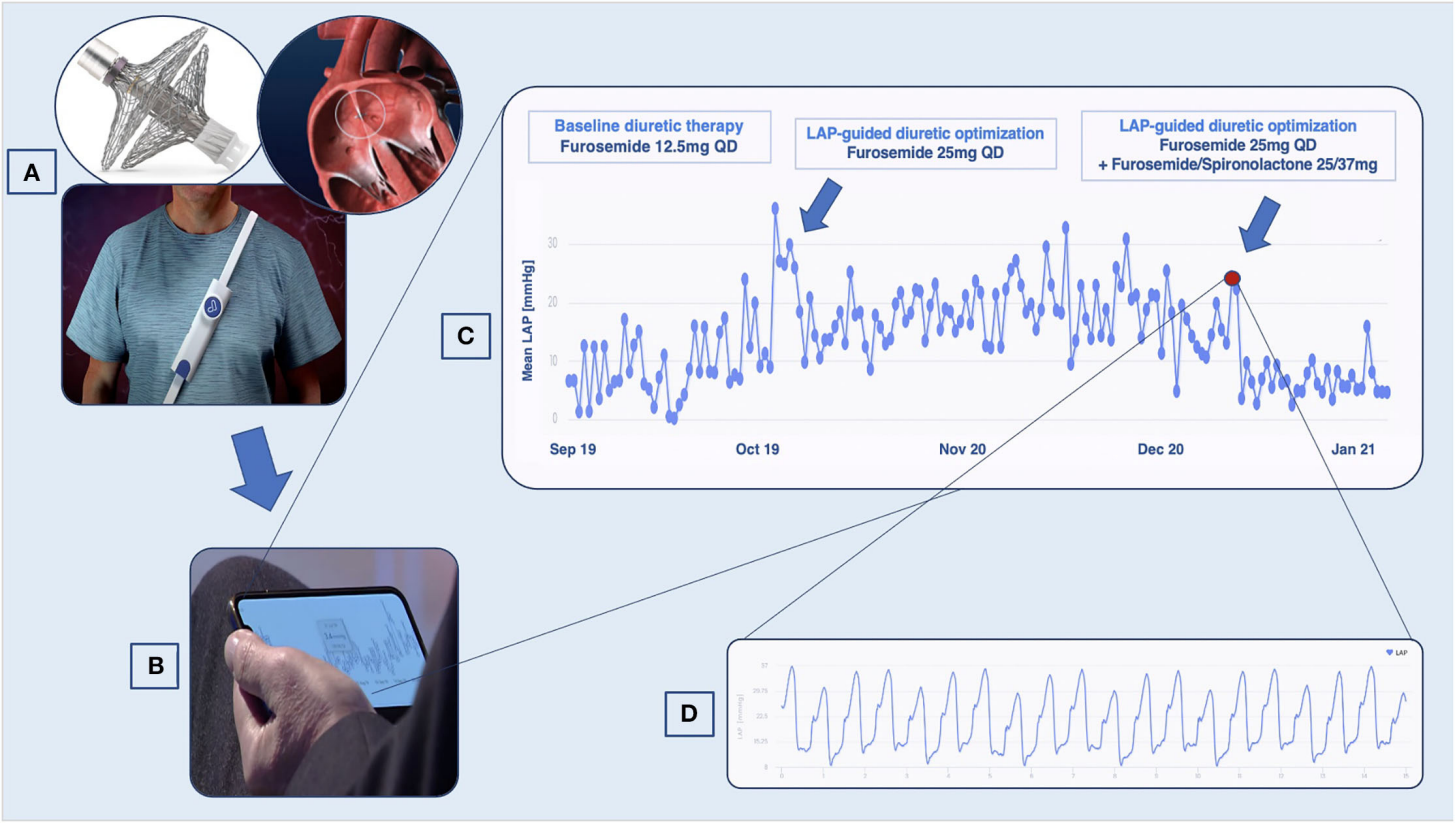
VLAP^TM^-empowered management of patients with heart failure (HF). **(A)** Once implanted through the interatrial septum, the device transmits left atrial pressure (LAP) data to an external system, which the patient wears once daily to perform the measurement. **(B)** Data are accessible to the cardiologist with a secured cloud-based system. **(C)** LAP trends showing variation suggestive of decompensation. **(D)** High-resolution LAP waveforms.

In our center, as part of the VECTOR-HF trial, V-LAP™ was implanted in five patients suffering from HF and in the NYHA III class. LAP trends have been remotely monitored over time to guide the optimization of therapy (Figure 1). The preliminary results concerning the usability and technical performance of the device have been already discussed elsewhere (12). The present manuscript aims to report the largest and longest single-center experience with this device in HF patients with a wide spectrum of HF presentations (Tables 1, 2).

**Table 1:** Clinical characteristics of the patients included.

**Variable**	**Patient 1**		**Patient 2**		**Patient 3**		**Patient 4**		**Patient 5**	
	**Enrollment**	**End of FU**	**Enrollment**	**End of FU**	**Enrollment**	**End of FU**	**Enrollment**	**End of FU**	**Enrollment**	**End of FU**
***Demographics and follow-up characteristics***										
Age (years)	75	77	71	73	67	68	72	73	76	76
Sex	male		male		male		male		female	
Length of FU (months)	32		23		18		15		3	
Daily adherence (%)	95		95		99		68		96	
Medical changes (n)	9		7		4		3		1	
***Risk factors, clinical history and presentation***										
Diabetes mellitus	no		yes		yes		no		yes	
Hypertension	no		yes		no		yes		yes	
CAD	no		yes		no		no		No	
AF	no		no		yes, permanent		yes, paroxysmal		yes, paroxysmal	
ICD	yes		yes		yes		yes		yes	
CRT	yes		no		yes		no		yes	
Admissions for HF (*n*) ^*^	2		1		1		2		3	
Predicted death (%) ^∞^	9 / 16		6 / 12		7 / 13		7 / 13		9 / 16	
NYHA Class	III	II	III	II	III	II	III	III	III	III
6-MWT (meters)	280	580	400	425	450	480	280	280	90	
KCCQ css / KCCQ oss	48 / 43	91 / 84	88 / 80	91 / 88	78 / 73	93 / 87	65 / 60	60 / 68	22 / 24	
***Drugs***										
BB	yes	yes	yes	Yes	yes	yes	yes	yes	yes	yes
ACE-I/ARBs/ARNi	yes	yes	yes	yes	yes	yes	yes	yes	yes	yes
MRAs	yes	yes	yes	yes	yes	yes	yes	yes	yes	yes
SGLT2i	no	yes	yes	yes	no	yes	no	no	no	no
Loop diuretic	yes	yes	yes	yes	yes	yes	yes	yes	yes	yes
Daily dose of loop diuretic	12.5 mg	37.5 mg	25 mg	50 mg	100 mg	150 mg	250 mg	250 mg	175 mg	175 mg
***Blood tests***										
Hemoglobin (mg/dl)	13.5	14.6	15.4	15	15.7	15.4	13.7	13.9	11.4	12
eGFR (ml/min/1.73 m^2^)	61	54	90	72	50	32	39	47	51	39
NT-proBNP (pg/ml)	1230	848	345	248	3131	2868	1932	2711	574	397

∞ *These data are referring to the 1- and 3-year predicted mortality risk, assessed by the Seattle HF model*.

*ACE-I/ARBs/ARNi, angiotensin-converting enzyme inhibitors; angiotensin II receptor blockers; angiotensin receptor-neprilysin inhibitor; AF, atrial fibrillation; BB, beta-blockers; CAD, coronary artery disease; CRT, cardiac resynchronization therapy; FU, follow up; ICD: implantable cardioverter defibrillator; eGFR, estimated glomerular filtration rate; KCCQ css, Kansas City cardiomyopathy questionnaire clinical summary score; KCCQ oss, Kansas City cardiomyopathy questionnaire overall summary score; LAP, left atrial pressure; MRA, mineral-corticoid antagonists; NT-proBNP, N-terminal pro brain natriuretic peptides; NYHA, New York heart association; QD, daily; EOD, every other day; SGLT2i, sodium/glucose cotransporter-2*.

∞ *These data are referring to the number of HF-related hospitalizations that occurred over the last year before the enrollment*.

**Table 2:** Echocardiographic features of the patients included.

**Variable**	**Patient 1**		**Patient 2**		**Patient 3**		**Patient 4**		**Patient 5**	
	**Enrollment**	**End of FU**	**Enrollment**	**End of FU**	**Enrollment**	**End of FU**	**Enrollment**	**End of FU**	**Enrollment**	**End of FU**
LVEDD (mm)	65	61	60	59	56	53	68	58	51	
LVEDVi (ml/m^2^)	114	112	117	118	91	81	91	74	88	
LVESVi (ml/m^2^)	85	88	77	79	66	57	58	48	62	
EF (%)	25	21	34	33	28	30	36	36	30	
Segments affected by WM abnormalities (n)^∞^	0	0	2	2	0	0	0	1	1	
Diastolic dysfunction	NA	NA	II	II	III	II	III	II	I	
LAVi (ml/m^2^)	53	44	29	35	60	39	65	61	25	
TAPSE (mm)	17	11	18	19	17	15	12	13	22	
PAPs (mmHg)	29	26	30	NA	55	38	40	NA	25	
MR	moderate^*^	mild^*^	mild	mild	moderate	mild	mild	mild	mild	
TR	mild	mild	mild	absent	mild	mild	mild	absent	mild	

*EF, ejection fraction; GLS, global longitudinal strain; IVC, inferior vena cava; LAD, left atrial diameter; LAVi, left atrial volume indexed; LVEDD, left ventricular end-diastolic diameter; LVEDV, left ventricular end-diastolic volume; LVESV, left ventricular end-systolic volume; MR, mitral regurgitation; NA, not assessable; PAPs, pulmonary artery systolic pressure; RVFAC, right ventricular fractional area change; TAPSE, tricuspid annular plane systolic excursion; TR, tricuspid regurgitation. WM, wall motion*.

∞ *Regional wall motion abnormalities were defined as akinesia or dyskinesia (ipokinesia was not included in the definition)*.

* *The patient had a prosthetic valve*.

## Results

### Patient 1

**Figure 2:**
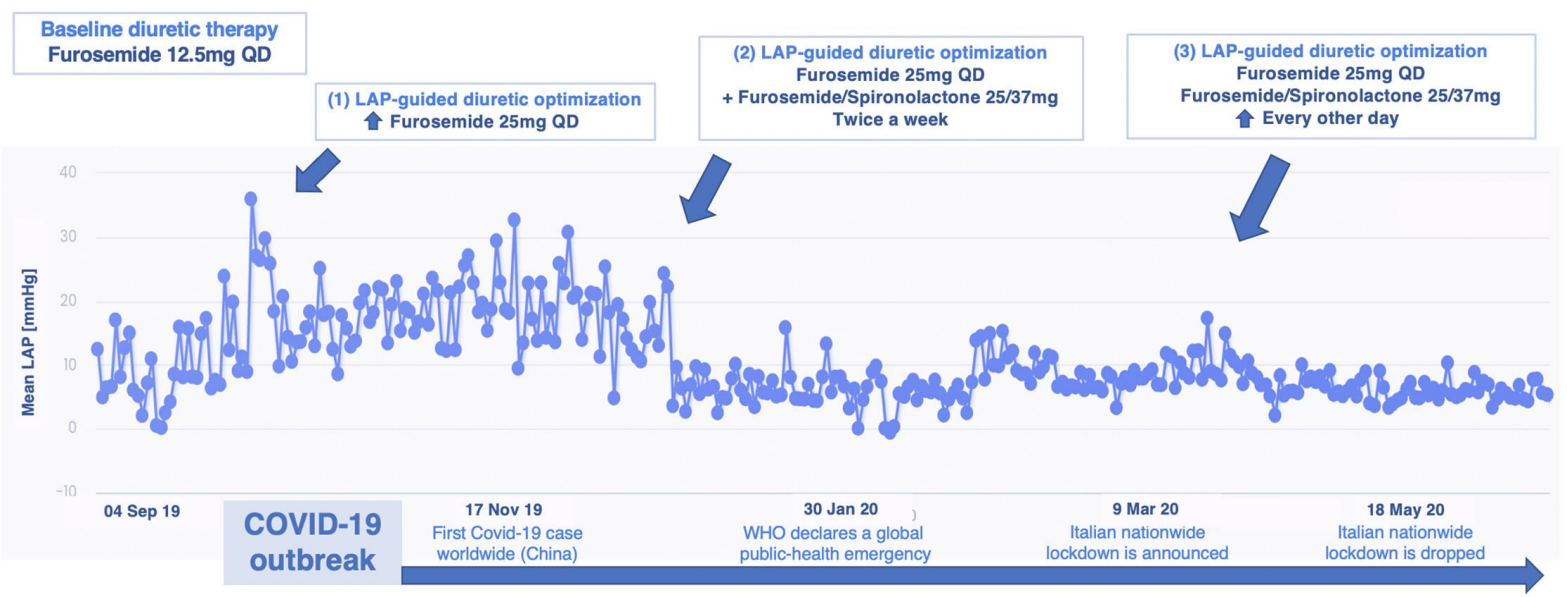
Remote LAP-guided therapy optimization over 12 months from VLAP™ implantation in patient 1: upper, therapy adjustments are reported; lower, milestones from the first coronavirus disease 2019 (COVID-19) wave are indicated. LAP, left atrial pressure; WHO, World Health Organization; QD, once daily.

This is the case of a 75 year-old male, suffering from HF with reduced ejection fraction (HFrEF) (LVEF 25%). The patient reported no history of coronary artery disease (CAD) and remained symptomatic for dyspnea on mild exertion (NYHA class III) despite optimal medical therapy (OMT) with beta-blockers, angiotensin receptor-neprilysin inhibitor (ARNi), mineralocorticoid receptor antagonist (MRA), loop diuretics plus device therapy with implantable cardioverter-defibrillator (ICD), and cardiac resynchronization therapy (CRT). His cardiovascular history started in 2015 when he was diagnosed with severe mitral regurgitation (MR), surgically treated with valve replacement. After V-LAP implantation and reliability confirmation, LAP measurements were analyzed. The patient reported high compliance (> 95%) with the measurements. In the first 12 months, a progressive increase in LAP was observed. Accordingly, the medical therapy was tailored, increasing the titration of furosemide and spironolactone, ranging from an initial daily dosage of furosemide of 12.5 mg every day (QD) to the current dose of furosemide/spironolactone 25/37 mg QD plus furosemide 25 mg every other day (EOD). The patient was remotely instructed to perform the proposed therapy adjustments and a stable LAP reduction was finally achieved (Figure 2). Over a total follow-up of 32 months, neither hospitalization nor urgent visits occurred. No further LAP-guided intervention was necessary over the 2nd year from implantation, observing sustained low intra-cardiac pressures. The reduction in LAP values was coupled with relief in symptoms (from NYHA class III to class II up to 30 months) and improvement in quality of life: the Kansas City Cardiomyopathy Questionnaire (KCCQ) overall summary score and clinically summary score raised from 42.7 and 47.9 at baseline to 84.4 and 90.6 at 24 months follow-up, respectively. Consistently, functional capacity ameliorated as the 6-min walking test (6MWT) improved from 280 m at baseline to 580 m at 24 months. The last recorded NT-proBNP value (848 pg/ml at 24 months visit) was persistently lower than the baseline (1,230 pg/ml).

### Patient 2

**Figure 3:**
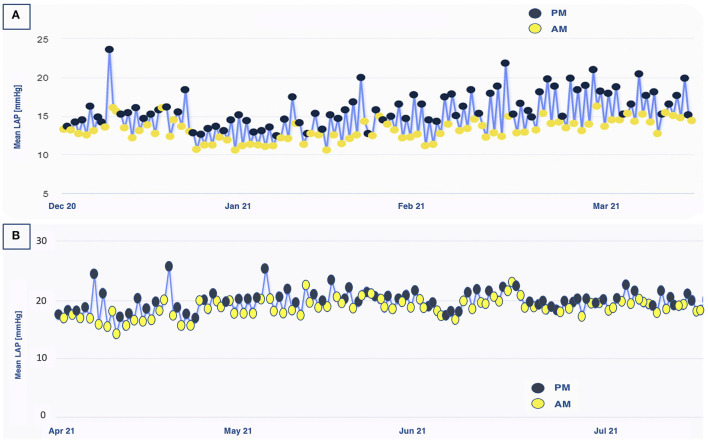
Left atrial pressure fluctuations throughout the day in patient 2. **(A)** Before diuretic dosage adjustment, a clear difference in PM/AM values was observed over a 3-month period. **(B)** After diuretic dosage adjustment, these hour-to-hour variations were blunted. AM, ante meridiem; PM, post meridiem; LAP, left atrial pressure.

A 71 year-old male patient suffering from ischemic chronic HFrEF (LVEF 34%), yet in OMT, experienced worsening dyspnea (NYHA III) and was hospitalized for decompensation. After discharge, the patient still reported mild exertion dyspnea and he was enrolled in the VECTOR-HF trial. Medical therapy has been consistently potentiated, according to the LAP trends recorded. The patient's tolerance to increased diuretic regimens was low, reporting hypotensive events and needing several furosemide daily dose adjustments over time. Guideline-oriented medical therapies (beta-blocker and ARNi) were further up-titrated. Although a clear LAP reduction was not observed, the slope of the rising trend was notably blunted, and the patient experienced no acute decompensation during follow-up. When the patient was solicited to double his daily LAP measurements, a constant discrepancy in LAP values throughout the day was revealed, with higher pressure being recorded in the evening hours. This trend was not explained by the normal hemodynamic circadian rhythm, featured by inverse diurnal rise. Diuretic dosage over the 24 h was recalibrated and these variations were flattened (Figure 3). To date, its data from LAP values are considered high in absolute terms (LAP > 20 mmHg). Symptoms were improved as he complained of dyspnea only on intense exertion (NHYA II) and no indication of hospitalization (or urgent visit) was reported during the entire follow-up period. Therefore, we assumed that the daily fluctuation and the trends over time, rather than the absolute values of LAP, might have an impact on prognosis. The quality of life evaluation models reflected this consideration: the KCCQ overall summary score raised from 79.69 at baseline to 88.54 at 12 months FUP and the KCCQ clinical summary score from 88.54 at baseline to 90.63 at 18 months FUP. The 6MWT demonstrated a slight functional improvement: from 400 m from baseline to 425 m at 18 months. In addition, a mild decrease in NT-proBNP value was noted (248 pg/ml), as compared with the baseline assessment (345 pg/ml).

### Patient 3

**Figure 4:**
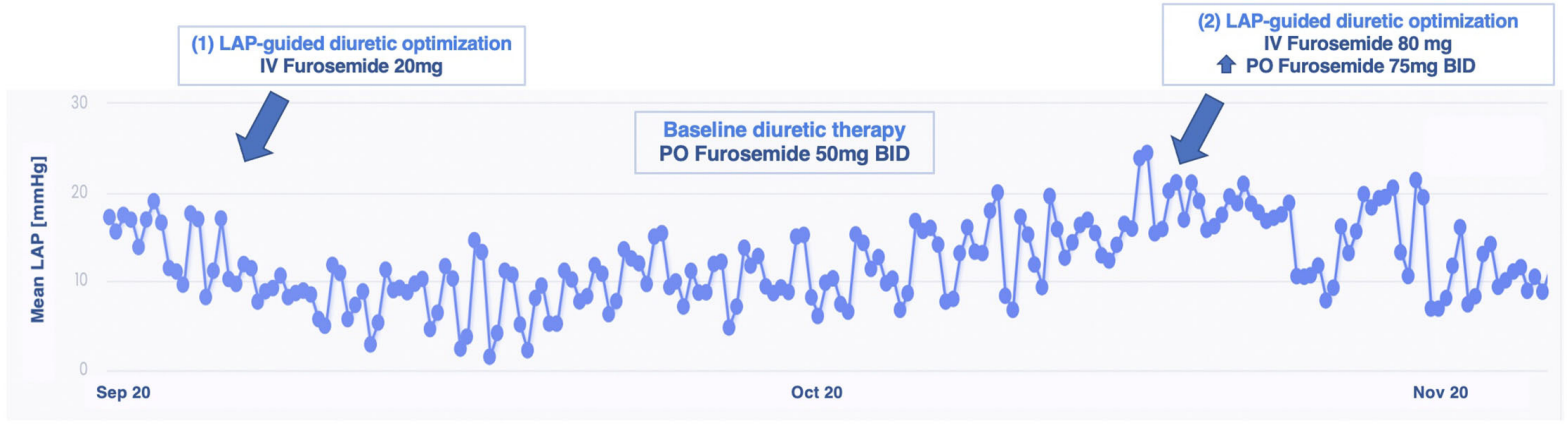
Left atrial pressure-guided IV diuretic administration managed to reduce LAP over time in patient 3. BID, bis in die; IV, intravenous; LAP, left atrial pressure; PO, per os.

A 67 year-old man with a long history of hypertrophic cardiomyopathy, with impaired systolic function (LVEF 28%), experienced multiple episodes of HF decompensation, despite OMT. Due to his advanced age, he was denied a heart transplant. After enrollment, we observed substantial LAP fluctuations in day-to-day measurements, hindering our aim to identify any gradual trend and estimate the risk of decompensation. Among our cohort, he is the only one with permanent atrial fibrillation, and the irregularity in his LAP trends should be, at least in part, explained by this condition. To overcome this disadvantage, we asked the patient to perform the measurements two times a day. He showed high compliance to daily LAP assessments, amounting to 99% at 18 months. Over almost a year, only two hot phases of sustained increasing LAP were noticed and treated with ambulatory intravenous diuretic therapy (up to furosemide 80 mg) plus the increase of the oral diuretic daily dosage. A LAP reduction was obtained, and no further decompensation was reported (Figure 4). During the entire follow-up, the patient referred clinical stability and symptomatic relief, preserving NYHA functional class II, and no further hospitalizations were needed. A functional mild improvement was reflected also in the 6MWT (ranging_from 450 m at baseline to 480 m at 18 months), in the KCCQ scores (from 73.18 and 77.60 at baseline to 87.06 and 93.18, for an overall and clinical summary score, respectively) and in NT-proBNP values (from 3,131 pg/ml at baseline to 2,868 pg/ml at most recent evaluation).

### Patient 4

A 72 year-old man with HFrEF (LVEF 36%) and non-ischemic dilated cardiomyopathy (LVEF 36%) was evaluated by our HF unit for dyspnea on mild exertion, peripheral edema, and increased NT-proBNP levels. Being ineligible for a heart transplant and unwilling to implant a left ventricular assist device, in November 2020, the choice was made to employ V-LAP remote monitoring. The adherence to daily LAP measurements was lower than other patients (65–70% at 15 months), impairing our ability to provide a personalized treatment. To date, he still complains of shortness of breath during daily activities (NYHA III), and an episode of sustained ventricular arrhythmia was recorded and treated by ICD with an appropriate shock. The available LAP measures did not display any reliable steady progression nor clear pathological elevation, but due to limited data, in-person assessments were scheduled every 2 months. Based on clinical findings, intravenous furosemide was administered during these appointments. A LAP reduction was observed and no hospitalization occurred. NT-proBNP levels increased from 1,932 pg/ml (prior implantation) to 2,711 pg/ml (at 12 months). As shown by KCCQ values, quality of life perception remained almost unchanged: the overall summary score ranged from 59.79 at baseline to 67.71 at 12 months, whereas the clinical summary score passed from 65.42 at baseline to 60.41 at 12 months. Comorbidities, such as chronic obstructive pulmonary disease and severe polyarticular osteoarthrosis could have affected his quality of life and physical capability, evaluated by the 6MWT, which reported poor performance (280 m without significant variation observed during the follow-up).

### Patient 5

A 76 year-old female patient, suffering from HFrEF (LVEF 30%) and non-ischemic dilated cardiomyopathy, experienced three hospitalizations for acute HF over 2021. She reported a very active lifestyle until a few months before, but in November 2021, the symptoms burden forced her home. Despite a complete up-titration of all guidelines-indicated medical therapies and CRT-D implantation, dyspnea on mild exertion persisted and the recurrence of peripheral edema required frequent diuretic dosage adjustments. In the absence of any feasible end-stage therapeutic option for this patient, she was implanted with the V-LAP monitoring system. At the end of February 2022, the calculated LAP was compared with the pulmonary capillary wedge pressure measured through right heart catheterization. Since then, LAP data have been visible to the clinical team. The extreme adherence (96%) to daily measurements over the first 3 months since implantation will help us to interpret future LAP trends and to avoid further HF exacerbations.

## Discussion

The V-LAP system represents an innovative implantable device for patients with advanced chronic HF aiming to reduce HF-related hospitalizations. A rise in LVEDP can be observed since the earliest phases of HF subclinical progression, offering a sweet spot to reduce intracardiac pressures and, in turn, prevent hospital readmission. The PAP monitoring system has not demonstrated a clear benefit on HF prognosis (6, 7). The mismatch between the right and the left heart pressures might be a possible explanation, and a sensor able to directly assess LAP could overcome these limitations.

The multicenter VECTOR-HF first-in-man trial is evaluating the safety, usability, and performance of the V-LAP™ system in those individuals who cannot benefit from the guidelines-indicated end-stage solution, being not eligible for heart transplant nor, in turn, for mechanical circulatory support.

In the present issue, we report our initial 3-year-experience with five patients with advanced HF, with a history of frequent hospital readmission and recurrently increased proBNP levels, showing the feasibility and safety of this approach.

Since the first human use of an implanted LAP monitoring system, trans-septal puncture has proved to be mechanically safe without neither cardiovascular nor neurological concerns (13, 14). However, both during the implantation and after the deployment of the interatrial device, the risk of thrombus formation and arterial side embolic complication is not negligible. To face this threat, according to VECTOR-HF protocol, dual antiplatelet therapy with clopidogrel plus aspirin was established before V-LAP implantation. Standard loading doses of both antiplatelet drugs were administered in naïve patients. During the procedure, unfractionated heparin was administered intravenously to achieve an activated clotting time of 250–350 s. A dual antiplatelet regimen was maintained for 3 months post-procedurally. In patients already on oral anticoagulants, the antithrombotic therapy requires the addition of a single antiplatelet agent. After 3 months, the therapy was transitioned to a single antiplatelet or anticoagulant agent, respectively. No periprocedural adverse events were observed in our 5 patients. At the time of the writing of this manuscript, all the individuals enrolled at our institution are free of major adverse cardiac and neurological events.

Importantly, the overall adherence of our patients to daily LAP measurements amounted to 87.6 ± 11.8%, attesting to remarkable patient usability and acceptance. These data confirm the optimal attitudes of patients toward V-LAP^TM^ usage that has been highlighted in a preliminary analysis by another center (15).

Over a cumulative follow-up time of 91 months, with a median follow-up time of 18 months, LAP trends-guided therapy optimization was accomplished. These hemodynamically driven adjustments succeeded in noticeably reducing LAP, thus preventing decompensation. No HF-related hospitalization occurred in all patients considered; functional capacity improved in three out of five patients (NYHA class from III to II) and was paralleled by an increase in the perceived quality of life, as indicated by the KCCQ, and physical capability assessed by 6MWT (as shown in Table 1).

Specifically, we scrutinized, daily, any asymptomatic gradual increase in LAP that might be a precursor for upcoming acute decompensation. When a significant stable variation was discovered, patients were instructed to modify their home therapy, primarily diuretics, obtaining a decline in LAP in most cases. Moreover, daily knowledge of LAP facilitated the up-titration of ACEi/ARBs, β-blockers, and ARNi, whose crucial influence on outcomes is well-established (16).

Among our subjects, we observed an important variability in LAP tolerance, reflecting different hemodynamic compensation. This supported our belief that hemodynamic remote monitoring should be focused on LAP progression rather than absolute LAP values, and no strict cut-off can be imposed to guide therapy optimization. At the same time, the need for stable trends makes it challenging to examine patients with irregular LAP fluctuations. Therefore, specific algorithms could be considered in the analysis of the LAP values in a specific class of patients with HF.

The V-LAP system also provides the cardiologists with additional critical information extracted from the analysis of the high-resolution LAP waveforms morphology. These accurate clues, when coming from the left chambers, may reveal both arrhythmias, such as atrial fibrillation by flattening of the atrial kick wave, and left-sided valvular defects, especially MR. Of note, PAP monitoring systems are unable to focus on mitral valve defects, whereas a good correlation has been proven between LAP and MR, as the latter highly impacts on LAP V-wave (17).

Overall, the lack of clinical HF exacerbations observed in our cohort of patients could be due, at least in part, to LAP-guided therapy management. This evidence supports the belief that personalized management in advanced HF can be the way forward.

The engagement of our patients with the device was very high, as proven by the daily adherence rates, which suggested remarkable reliability and ease of use of the system. In this regard, we learned from our cases that trusty LAP trends require constant data, and the more the patient is compliant with daily measurements, the easier we can realize proactive management to anticipate any acute decompensation. For this reason, an accurate selection of patients seems crucial when dealing with remote hemodynamic monitoring.

Another secondary application of V-LAP emerged when we asked our most compliant patients to take measurements more than once daily. The occurrence of intraday remarkable differences in LAP measurements, inconsistent with the physiological variations due to the circadian rhythm, was addressed by optimizing the time and dosage of drug assumption.

The potential of chronic HF management through remote monitoring of LAP has emerged during the coronavirus disease 2019 (COVID-19) pandemic (18). Since the Italian outbreak, V-LAP™ allowed us to keep the patients away from unsafe medical contacts and optimize the treatment while avoiding dangerous access to the hospital (19). The paucity of alternative healthcare resources during the pandemic has highlighted the importance of safer but still clinically appropriate solutions for those chronic diseases, such as HF, requiring constant surveillance. Apart from the COVID-19 challenging scenario, home monitoring devices have well-shown their reliability in general practice (6, 20, 21), and the time is ripe for a logistic paradigm shift in the management of patients with HF: telemonitoring cannot be seen anymore as a gain of distance, but rather as an enhancement of cardiac care and a reinforcement of the physician-patient privilege (22). Hence facilitating the mandatory transition toward telehealth as a way to warrant effective, sustainable HF-related care, even the COVID-19 clouds unveiled a silver lining.

## Conclusion

The application of the V-LAP system seems safe and reliable. In addition, preliminary clinical insights from our center's triennial experience support a promising efficacy of V-LAP empowered remote monitoring strategy in patients with advanced HF. However, future large trials will be crucial, both to confirm the clinical value of left-sided hemodynamic monitoring, and to standardize an effective LAP-guided medical management.

## Data Availability Statement

The original contributions presented in the study are included in the article/supplementary material, further inquiries can be directed to the corresponding author.

## Ethics Statement

The studies involving human participants were reviewed and approved by Fondazione Policlinico Universitario A. Gemelli IRCCS-Ethics Committee. The patients/participants provided their written informed consent to participate in this study. Written informed consent was obtained from the individual(s) for the publication of any potentially identifiable images or data included in this article.

## Author Contributions

FC, DD'A, and AR conducted the study. DP, FB, CT, GL, NA, GP, and RL supported the realization of the study. AR had a leading role in writing the manuscript. FC and DD'A had a leading role in manuscript revision. DP, LC, MG, and AI had a supporting role in manuscript revision. All authors have read and agreed to the content of the manuscript.

## Funding

Funding for the VECTOR-HF trial was made available by Vectorious Medical Technologies.

## Conflict of Interest

The authors declare that the research was conducted in the absence of any commercial or financial relationships that could be construed as a potential conflict of interest.

## Publisher's Note

All claims expressed in this article are solely those of the authors and do not necessarily represent those of their affiliated organizations, or those of the publisher, the editors and the reviewers. Any product that may be evaluated in this article, or claim that may be made by its manufacturer, is not guaranteed or endorsed by the publisher.

## Abbreviations

HF: Heart Failure
HFrEF: Heart Failure with Reduced Ejection Fraction
NYHA: New York Heart Association
ESC: European Society of Cardiology
LVEF: Left Ventricular Ejection Fraction
LVEDP: Left Ventricular End Diastolic Pressure
PAP: Pulmonary Artery Pressure
LAP: Left Atrial Pressure
PCWP: Pulmonary Capillary Wedge Pressure
OMT: Optimal Medical Therapy
ARNi: Angiotensin Receptor-Neprilysin inhibitor
ACEi/ARBs: ACE inhibitors/ Angiotensin Receptor Blockers
MR: Mitral regurgitation
MRA: Mineralocorticoid receptor antagonist
SGLT2i: Sodium-Glucose co-transporter 2 inhibitor
ICD: Implantable Cardioverter Defibrillator
CRT-D: Cardiac Resynchronization Therapy Defibrillator
KCCQ: Kansas City Cardiomyopathy Questionnaire
6MWT: 6 Minute Walking Test
NT-proBNP: NT-pro B-type natriuretic peptide.
